# Correlation of Histopathology and Radiological Findings Among the Diverse Breast Lesions in a Tertiary Care Centre

**DOI:** 10.7759/cureus.52097

**Published:** 2024-01-11

**Authors:** Ranjani Mohan, Sathish Selvakumar A, Ragupathy S, Meenakshisundaram K, Shanmugapriya S, Rajeswari Kathiah, Rajeswari T, Priavadhana Rajan Prasaad, Dinesh Kumar S, Sarika K

**Affiliations:** 1 Pathology, ESIC Medical College and Hospital, Chennai, IND; 2 Radiology, ESIC Medical College and Hospital, Chennai, IND; 3 Pathology, All India Institute of Medical Sciences, Madurai, IND

**Keywords:** breast diseases, ultrasonogram, fine needle aspiration, cancer screening, biopsy

## Abstract

Introduction

Breast lumps in any age group are addressed cautiously to exclude the possibility of breast cancer. The clinical approach to breast lumps involves the "triple test" for cancer screening. The triple test includes clinical examination, imaging (mammogram or ultrasonogram), and tissue sampling (fine needle aspiration cytology (FNAC) and core needle biopsy). These tests happen in a sequential process, and it is important that their findings support the final diagnosis for accurate management of the patient.

Aims and objectives

This study aims to determine the correlation between the histopathological and radiological findings among the various breast lesions and describe the spectrum of breast lesions received in our center.

Methods

This is a retrospective observational study for a period of three years, from January 2020 to December 2022. The study included 400 patients who had undergone ultrasonography or mammograms for breast lumps, FNAC, core needle biopsy, or surgical resection. The data collected was analyzed for concordance and discordance status.

Results

A total of 400 cases were reviewed. There were 238 (59.5%) histologically confirmed benign breast lesions and 162 (40.5%) malignant lesions with their corresponding BI-RADS (Breast Imaging Reporting and Data System) scores. The sensitivity, specificity, positive predictive value, negative predictive value, and diagnostic accuracy for the imaging modalities (ultrasonogram and mammogram) in diagnosing breast lesions were 95.06%, 94.96%, 92.77%, 96.58%, and 95%, respectively, which were comparable with other similar studies. The biological and immunohistochemical factors of all the invasive carcinomas were studied in detail.

Conclusions

Imaging modalities (ultrasonogram or mammogram) have good sensitivity and specificity in diagnosing breast lesions and can be reliably used as a preliminary test in breast lump evaluation. The BI-RADS score is a reliable indicator and can be considered for the effective follow-up or intervention of the breast lesion. In discordant cases, a repeated core needle biopsy or excision has to be recommended, as pathological diagnosis is the cornerstone of effective management. A good rapport between the surgeon, radiologist, and pathologist aids in effective feedback and learning for achieving diagnostic accuracy.

## Introduction

Breast cancer is the most common malignancy in females, with a global incidence of 2.26 million in 2020 [1]. The projected incidence of breast cancer is estimated at 2.7 million annually by 2030 [1]. Breast cancer is also the leading cause of cancer deaths among women [1]. Worldwide, breast cancer is responsible for deaths at an age-adjusted rate of 13.6/100000 [1]. Indian statistics show that the age-adjusted incidence rate in Chennai is 37.9/100000 women [2]. The increasing trend in the incidence of breast cancer worldwide mandates that every woman who presents with a breast lump must be subjected to the triple test for early detection of cancer and its management. Breast carcinoma detected in its early stages has a good prognosis with the advent of neoadjuvant chemotherapy tailored to the hormone receptor status, followed by surgery [1]. This signifies the great importance of each step of the triple test, clinical examination, imaging, and cytological and/or histopathological diagnosis of the breast lump, to provide corroborative diagnostic evidence for choosing the right clinical action.

After the clinical assessment, a breast lump is assessed radiologically by an ultrasonogram and/or mammogram, or rarely by an MRI or PET-CT scan. BI-RADS (Breast Imaging Reporting and Data System) characterizes the radiological findings and categorizes breast lesions into six groups for easy clinical understanding. This is a risk assessment tool and quality indicator developed by the American College of Radiology [3]. Following imaging, fine needle aspiration cytology (FNAC) or a core needle biopsy is performed for a definite pathological diagnosis. Discordance may arise due to sampling error, the diverse nature of the lesion, or interpretation error. A discordance rate of 1-8% for imaging and pathological diagnosis has been reported, of which 24% turned out to be malignant [3]. The primary aim of the study was to evaluate the frequency of radio-pathological discordance. Our secondary aim was to detail our findings regarding the spectrum of breast lesions diagnosed in our department.

## Materials and methods

This is a retrospective observational study conducted between January 2020 and December 2022 after the approval of the Institutional Ethics Committee (IEC/2023/1/04, dated February 22, 2023). Informed consent was obtained, and data from 400 patients from the medical records was collected. The data collected includes patient demographic data, clinical diagnosis, BI-RADS category for imaging (ultrasonogram or mammogram), FNAC and/or core needle biopsy diagnosis where available, and resection specimen details according to the College of American Pathologists (CAP) Protocol for reporting breast carcinoma.

Patients of all ages and both sexes with the BI-RADS category in their imaging report with a core needle biopsy investigation and/or surgically excised samples were included in the study. Patients with no BI-RADS category, patients with no FNAC/histopathology follow-up at our center, and biopsy cases with inconclusive results were excluded from the study.

The BI-RADS assessment categories are BI-RADS 0 (incomplete), which needs additional imaging or comparison with prior examinations; BIRADS 1 (negative) and BIRADS 2 (benign), for which routine mammography screening is recommended; BIRADS 3 (probably benign), for which follow-up every six months is recommended; BIRADS 4 (4A: low, 4B: medium, and 4C: marked suspicion of malignancy) and BIRADS 5 (highly suggestive of malignancy), for which tissue diagnosis is recommended; and BIRADS 6 (known biopsy-proven malignancy), for which surgical resection is recommended as per clinical indication [4].

Core needle biopsies were usually done for BI-RADS 4 and above lesions for a definite diagnosis and immunohistochemistry for selecting neoadjuvant chemotherapy for malignancies. BI-RADS categories 2, 3, and 4A were considered benign, and categories 4B, 4C, and 5 were considered malignant in this study. The FNAC diagnosis is categorized using the Yokohama system for reporting breast fine needle aspiration biopsy cytopathology [5]. The categories include category 1: insufficient material; category 2: benign; category 3: atypical, probably benign; category 4: suspicious of malignancy; and category 5: malignant [5].

FNAC was done using a 23-gauge blue needle in our department. Two to six slides were prepared and stained with hematoxylin and eosin, May Grunwald Giemsa, and Papanicolaou stains. We received core needle biopsy, excision, and MRM specimens in a labeled container with 10% neutral buffered formalin fixative, which was then taken for grossing, routine processing, and staining with hematoxylin and eosin. Immunohistochemistry was also examined and interpreted in all cases. Immunohistochemistry used was ER (Clone QR013, Quartett, ready-to-use), PR (Quartett), HER2 (Clone QR003, Quartett), and Ki67 (Clone-QR015, Quartett). Immunohistochemical findings of breast carcinomas in the study were noted.

Discordance was analyzed between imaging and pathological diagnosis. Findings from the BI-RADS category and the pathological diagnosis were tabulated, and concordance or discordance was observed. Sensitivity was calculated using the formula: TP/TP+FN; specificity: TN/FP+TN; positive predictive value: TP/TP+FP; negative predictive value: TN/TN+FN; and diagnostic accuracy: TP+TN/TP+FP+FN+TN (TP: true positive; TN: true negative; FN: false negative; and FP: false positive).

## Results

**Figure 1:**
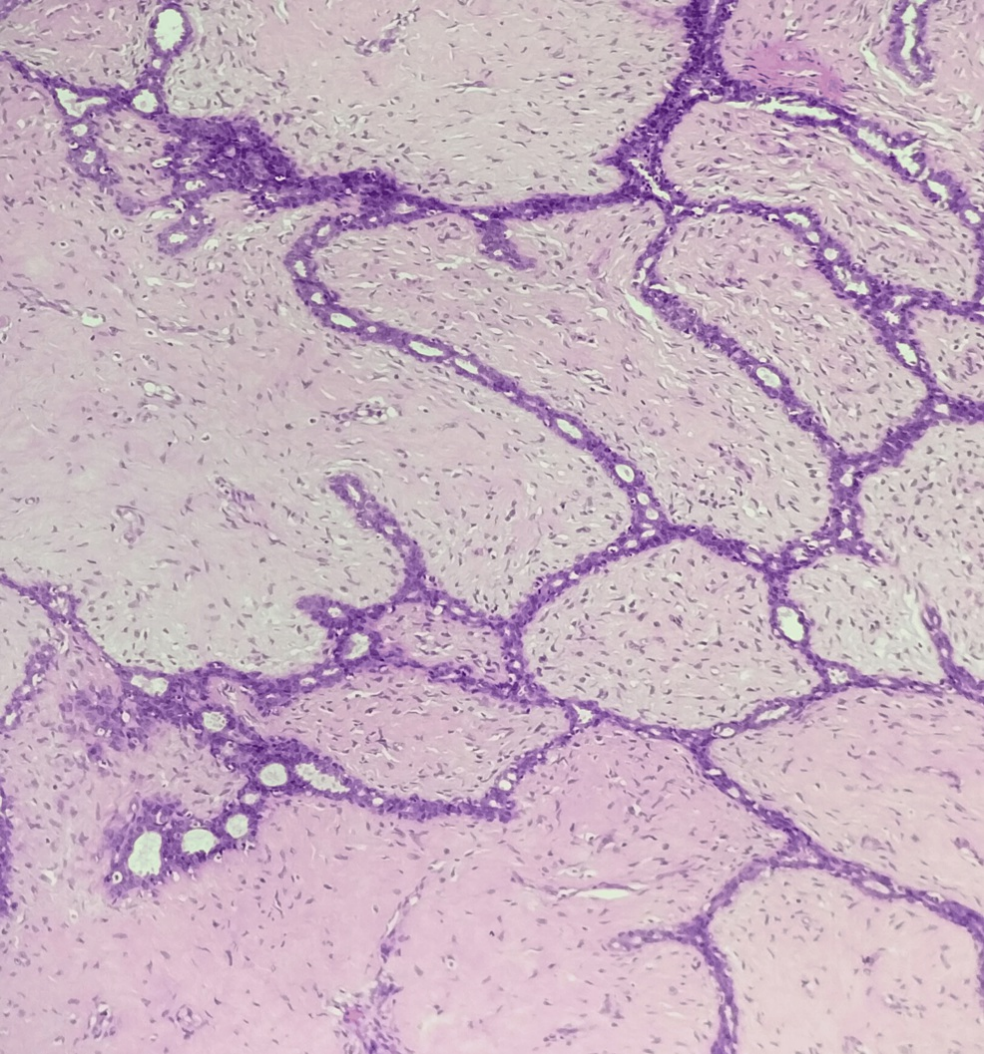
Fibroadenoma, hematoxylin and eosin stain, 100× magnification

**Figure 2:**
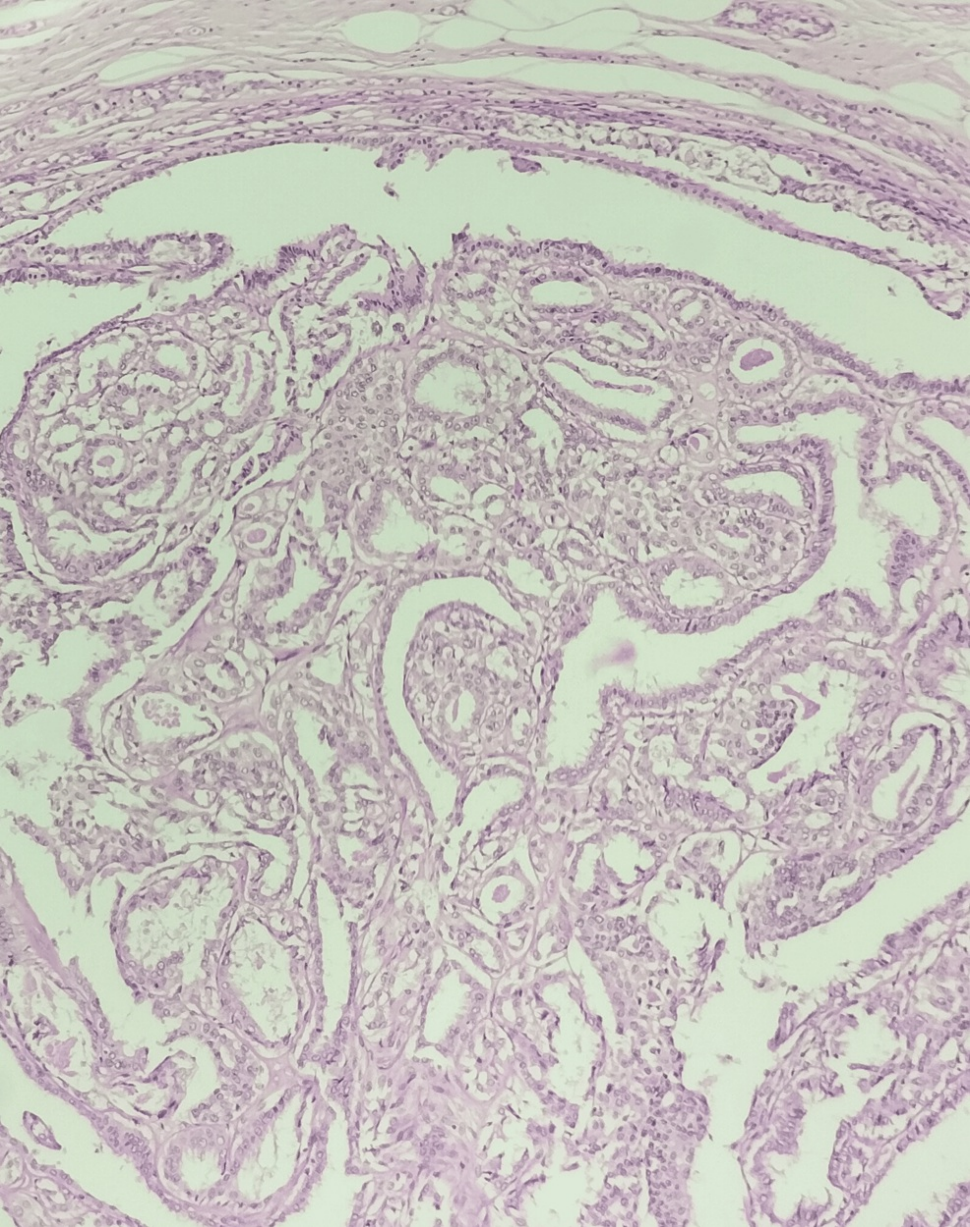
Intraductal papilloma, hematoxylin and eosin stain, 100× magnification

**Figure 3:**
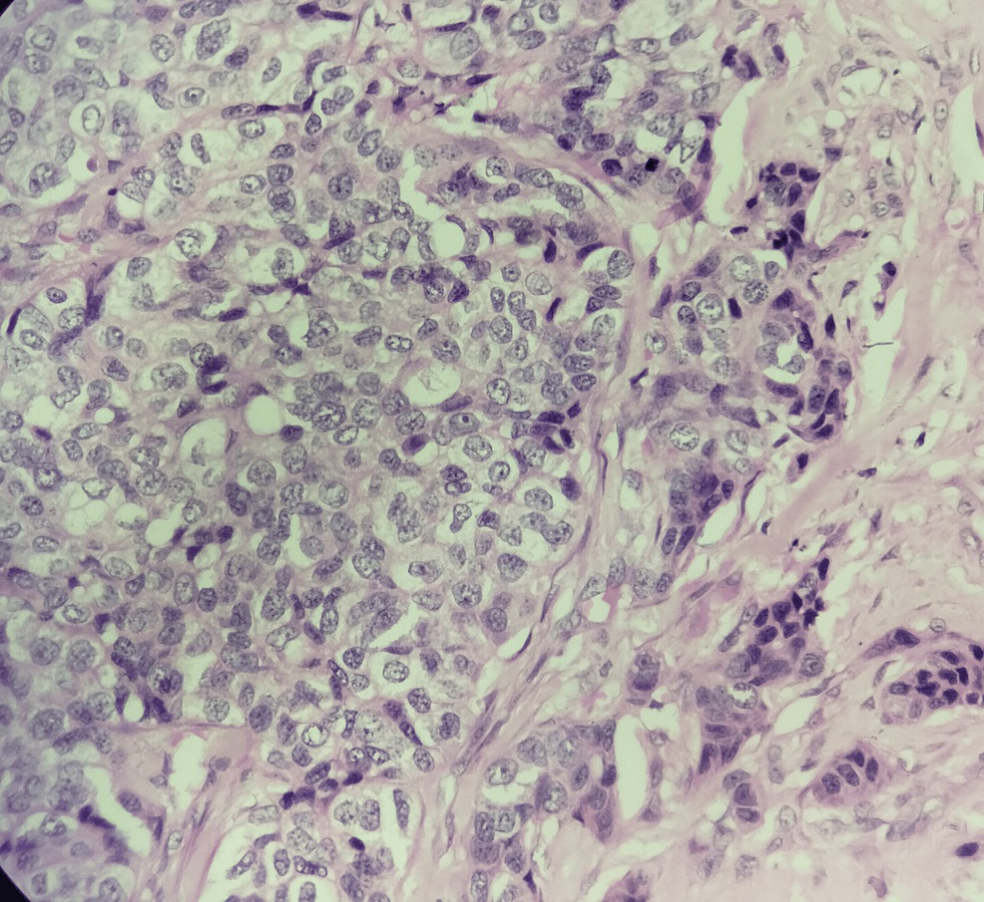
Invasive carcinoma, no special type, hematoxylin and eosin stain, 400× magnification

The study included 400 subjects, including 388 (97%) females and 12 (3%) males. All the male patients in the study had gynecomastia. The age range of male patients was 21 to 48 years. A total of 226 (58.25%) female patients had a non-neoplastic or benign breast lesion, and 162 (41.75%) females had an atypical or malignant breast lesion. The most common diagnoses in the benign category were fibroadenoma, fibrocystic change, and benign phyllodes tumor (Table 1; Figure 1). A single case of intraductal papilloma, among others, was reported during the study period (Figure 2). The most common diagnoses in the malignant category were invasive carcinoma (no special type), ductal carcinoma in situ (DCIS), and lobular carcinoma (Table 2; Figure 3).

**Table 1:** Distribution of non-neoplastic and benign breast lesions in the study

S.no	Non-neoplastic and benign lesions	Number of cases (total=238 )	Percentage % (n=400)
1.	Fibroadenoma	160	40
2.	Fibrocystic change	30	7.5
3.	Benign phyllodes tumor	10	2.5
4.	Breast abscess	15	3.75
5.	Granulomatous mastitis	2	0.5
6.	Sclerosing adenosis	3	0.75
7.	Usual ductal hyperplasia	2	0.5
8.	Intraductal papilloma	1	0.25
9.	Cavernous hemangioma	1	0.25
10.	Adenomyoepithelioma	1	0.25
11.	Columnar cell hyperplasia	1	0.25
12.	Gynecomastia	12	3

**Table 2:** Distribution of atypical/malignant breast lesions in the study DCIS, ductal carcinoma in situ

S.no	Atypical/ malignant lesions	Number of cases (total=162 )	Percentage % (n=400)
1.	DCIS	6	1.5
2.	Atypical ductal hyperplasia	2	0.5
3.	Borderline phyllodes tumor	2	0.5
4.	Malignant phyllodes tumor	1	0.25
3.	Invasive carcinoma, no special type	140	35
4.	Invasive lobular carcinoma	3	0.75
5.	Mucinous carcinoma	3	0.75
6.	Invasive carcinoma with medullary features	2	0.5
7.	Metaplastic carcinoma	1	0.25
8.	Invasive carcinoma, no special type with Paget's disease	1	0.25
9.	Diffuse large B-cell lymphoma	1	0.25

The age range of benign breast lesions was 13-68 years, and the maximum cases were between the ages of 20 and 30 years. Malignant breast lesions were reported in the age range of 24-80 years, with a mean of 55 years. 

The pathological samples studied include FNAC, core needle biopsy, and resection specimens. There were 238 confirmed non-neoplastic or benign cases and 162 malignant cases (a total of 400).

Concordant benign

The breast lesions categorized as BI-RADS 2 or 3 or 4A that showed a corresponding benign histopathological diagnosis were considered concordantly benign [6]. There were 226 cases (94.96%) (Table 3).

**Table 3:** Histopathological and radiological concordance and discordance

Histopathology	BI-RADS 2	BI-RADS 3	BI-RADS 4A	BI-RADS 4B/4C	BI-RADS 5	BI-RADS 6	Total
Non neoplastic/benign	52	169	5	9	3	-	238
Atypical/malignant	4	2	2	64	90	-	162
Total	56	171	7	73	93	-	400

Discordant benign

The breast lesions categorized as BI-RADS 4B/4C or BI-RADS 5 that showed a corresponding benign histopathological diagnosis were considered discordantly benign [6]. Twelve (5.04%) breast lesions categorized as BI-RADS 4B/4C or BI-RADS 5 were benign lesions on histopathological diagnosis (Table 3).

Concordant malignant

The breast lesions categorized as BI-RADS 4B/4C or BI-RADS 5 that showed a corresponding malignant histopathological diagnosis were considered concordant malignant [6]. A total of 154 (95.06%) breast lesions categorized as BI-RADS 4B/4C or BI-RADS 5 showed a corresponding atypical or malignant histopathological diagnosis (Table 3).

Discordant malignant

The breast lesions categorized as BI-RADS 2 or 3 or 4A that showed malignant histopathological diagnosis were considered discordant malignant [6]. Four cases (2.47%) in the BI-RADS 2 category turned out to be malignant on histopathology. Two (1.23%) cases with BI-RADS category 3 and 2 (1.23%) cases with BI-RADS category 4A were malignant on histopathological diagnosis (Table 3).

The sensitivity of the imaging modalities (ultrasonogram and mammogram) in diagnosing breast lesions was 95.06%. The specificity, positive predictive value, negative predictive value, and diagnostic accuracy were 94.96%, 92.77%, 96.58%, and 95%, respectively.

One patient with invasive carcinoma, no special type, had a history of ovarian serous carcinoma six months ago, for which she had undergone neoadjuvant chemotherapy and radical hysterectomy. BRCA status was not known for this patient at the time of this study. Only four cases of locally invasive breast carcinomas had undergone PET-CT scans.

**Figure 4:**
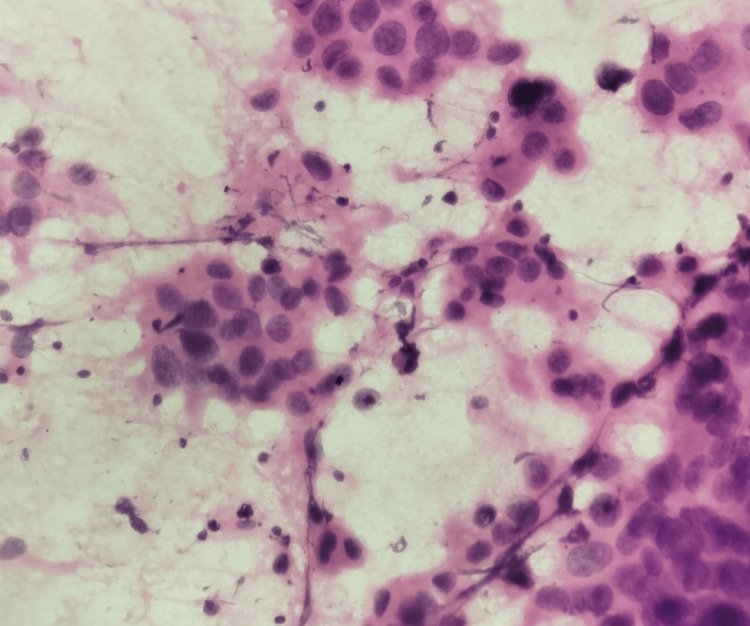
FNAC image of carcinoma breast, hematoxylin and eosin stain, 100× magnification FNAC, fine needle aspiration cytology

Among the study cases (n=400), 320 (80%) had a corresponding FNAC done. A total of 202 (63.13%) cases were reported as benign, and 118 (36.87%) cases were reported as atypical, suspicious, or malignant (Figure 4).

Six (1.8%) cases showed a discordant diagnosis compared to a histopathological diagnosis. Two breast lesions diagnosed as benign proliferative breast disease with atypia were fibroadenoma with epitheliosis in histopathology. Two breast lesions diagnosed as fibroadenoma by cytology were in fact invasive carcinomas, no special type in histopathology. One case diagnosed as fibroadenoma by cytology was a malignant phyllodes tumor, and one case diagnosed as fibroadenoma was a borderline phyllodes tumor on histopathology. The sensitivity, specificity, positive predictive value, negative predictive value, and diagnostic accuracy were 96.6%, 99%, 98.3%, 98%, and 98%, respectively. These six cases were radiologically discordant as well.

The study included 198 specimens of core needle biopsies, 168 excision specimens, and 34 specimens of modified radical mastectomy (MRM) with axillary lymph node dissection.

Out of 34 MRM cases done for carcinomas, 22 (64.7%) had the T2 stage, followed by T3 (11.76%, n=4), T1c (11.76%, n=4), T0 (8.82%, n=3), and T4b (2.94%, n=1) stages. Nodal status was predominantly N0 in 50% (n=17) cases, N1a in 32.3% (n=11) cases, N2a in 11.8% (n=4), and Nx in 5.8% (n=2) of the cases. Five to 28 lymph nodes were sampled in the MRM specimens, with an average number of 14 lymph nodes. Metastasis cannot be assessed in all cases. These data indicate that most women seek medical attention and treatment when the tumor size is between 2 cm and 5 cm (T2 stage).

Nottingham histologic grade was assigned to all the invasive carcinomas (n=150). Eighty-two (54.6%) tumors belonged to Nottingham histologic grade II. Forty-eight (32%) tumors belonged to grade III, and 20 (13.3%) tumors belonged to grade I.

**Figure 5:**
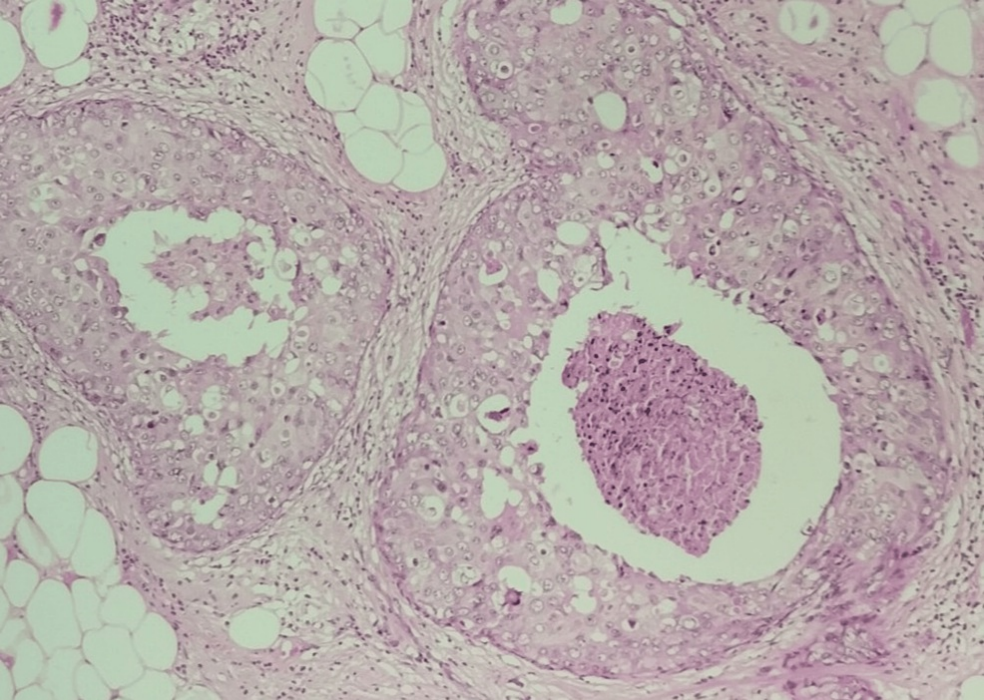
DCIS with comedonecrosis, hematoxylin and eosin stain, 100× magnification DCIS, ductal carcinoma in situ

DCIS was noted as an additional finding in 11.76% (n=4) of MRM specimens. The patterns of DCIS seen were solid, cribriform, micropapillary with central necrosis, and comedonecrosis (Figure 5).

Fourteen specimens of MRM went through post-neoadjuvant chemotherapy. The Miller-Payne grading system for assessing response to neoadjuvant chemotherapy showed grade 2 response in 42.86% (n=6) of cases, complete response (grade 5) in 35.7% (n=5) cases, grade 4 in 14.29% (n=2) cases, and grade 3 in 7.14% (n=1) cases.

A frozen section was sought for 2 cases of MRM specimens to ascertain margin status, and both cases showed negative margins.

**Figure 6:**
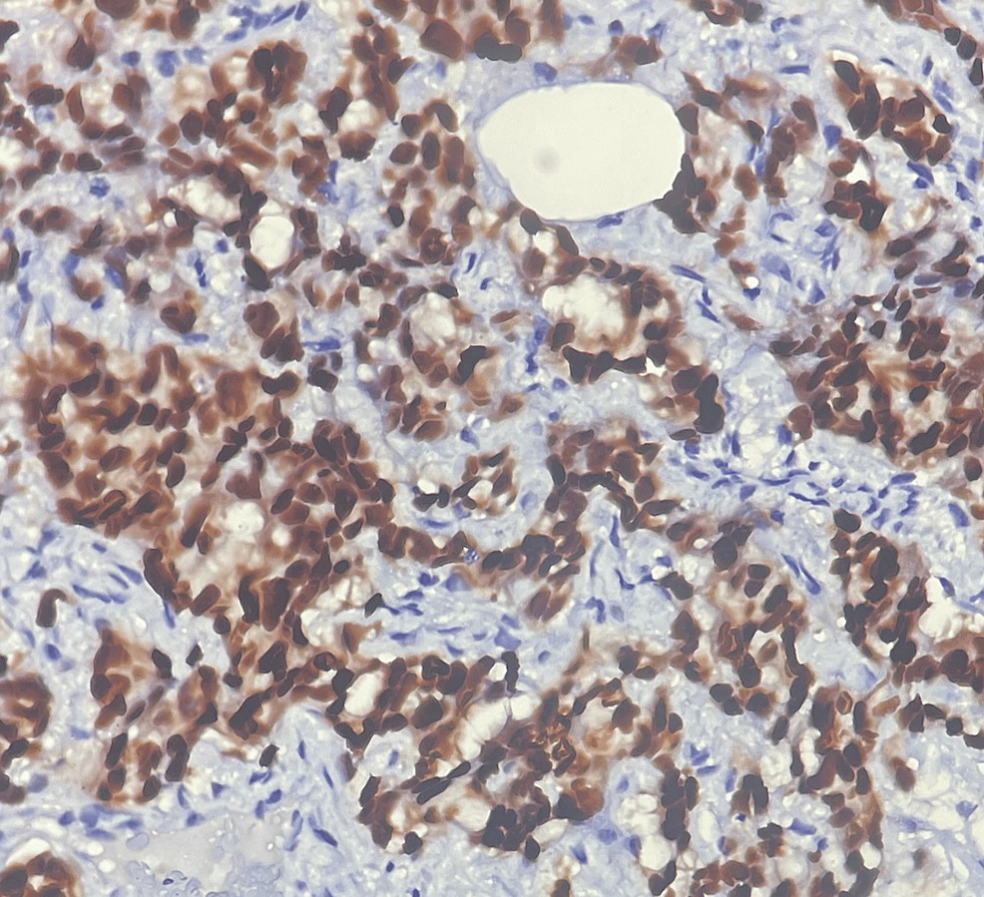
ER positive. Allred score: 5+3=8, immunohistochemistry, 400× magnification Allred score = proportion score (5) + intensity score (3) = total score (8).

**Figure 7:**
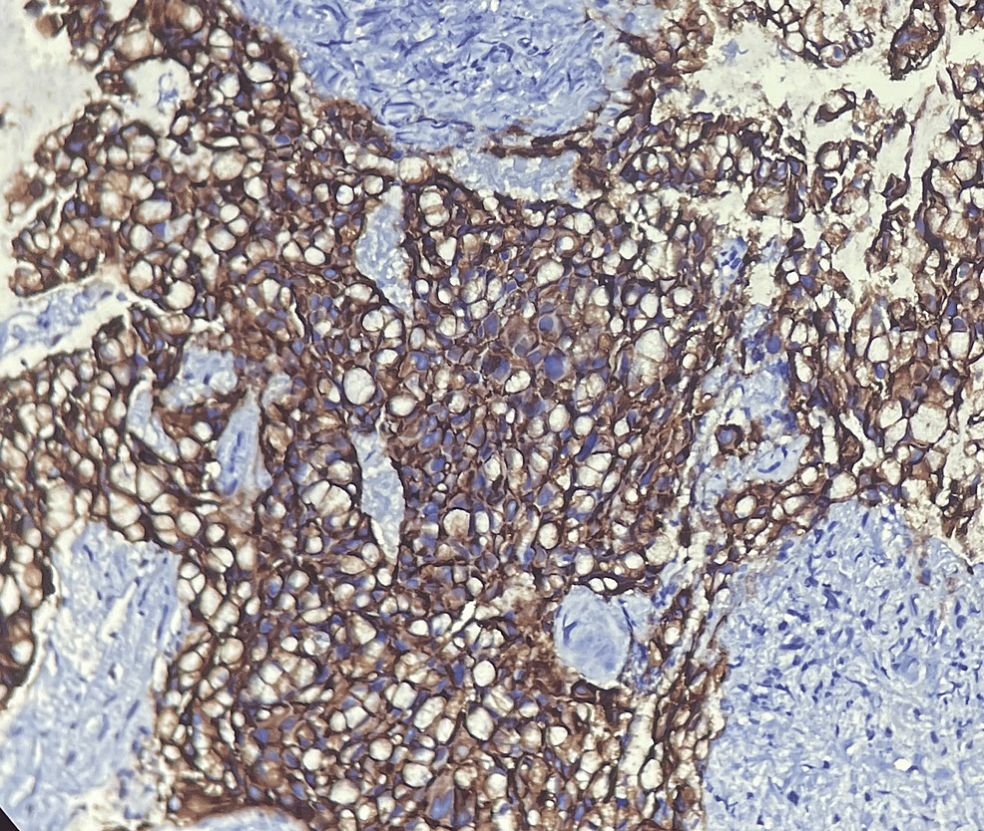
HER2 score: 3+ (positive), immunohistochemistry, 100× magnification HER2 score: 3+ is a strong complete membrane staining in more than 30% of tumor cells.

Immunohistochemistry for the hormonal status of ER, PR, Her2neu, and Ki67 was performed on 156 invasive carcinomas and DCIS. ER-positive and HER2-negative tumors were 55 in number (34.8%). ER, PR, and HER2-negative tumors were 50 in number (31.6%), ER-positive and HER2-positive tumors were 28 in number (17.7%), and HER2-enriched tumors were 25 in number (15.8%) (Figure 6; Figure 7). The Ki67 index for all the tumors ranged from 0% to 90%.

## Discussion

BI-RADS, initially released in 1993, was revised (5th) in 2014 by the American College of Radiology. This was framed to clearly communicate the radiological findings in breast lesions with uniformity and clarity to the treating physicians. This also serves as a quality assurance tool. Similar lexicons are available for lung cancer screening (Lung-RADS), head injury (HI-RADS), liver (LI-RADS), and prostate cancer (PI-RADS) [4].

Similarly, FNAC has a reporting system to give clear information about risk to clinicians. In our study, the comparison of FNAC results with histopathology results was done, and the results were similar to other studies by Poornima V. Kamatar et al., Sana Ahuja et al., Archana Sagar et al., and Gulcin Ozkan Onur et al. [5,7-9].

The results obtained in the comparison of BI-RADS with the pathological diagnosis were comparable with similar studies like Archana et al., Jitendra Parmar et al., Lailla Carolline et al., Ji Hyun Youk et al., Vinod Kumar et al., and Renato de Oliveira Pereira et al. [6,10-14]. Jitendra Parmar et al. recorded sensitivity, specificity, positive predictive value, negative predictive value, and diagnostic accuracy of 97.7%, 95.6%, 91.5%, 98.9%, and 96.3%, respectively [11]. Archana et al. recorded sensitivity, specificity, and diagnostic accuracy of 85%, 96%, and 90%, respectively [10]. Lailla Caroline et al. showed a 97% of cases with BI-RADS 4A had a benign histopathological diagnosis and all cases with high suspicion of malignancy had a malignant histopathological diagnosis [12]. A BI-RADS 3 lesion has a 2% or lesser probability of malignancy and hence can be followed up with short-term imaging surveillance [6].

Benign breast lesions show a well-circumscribed margin on an ultrasonogram or mammogram with dot-like calcifications at the periphery of the lesion or popcorn-type calcification in the entire lesion. Phyllodes tumor is usually a large iso-dense mass in imaging with plaque-like calcification [15]. On imaging, malignant breast lesions show irregular margins and fine linear or fine branching calcification.

On core needle biopsy, distinguishing malignant phyllode tumors from spindle cell metaplastic carcinoma may pose a challenge [16]. The upper outer quadrant is the most frequent site for breast cancer. This finding was comparable to similar studies by Archana Sagar et al. [8].

The Nottingham histologic criteria for grading breast carcinomas take into account the percent of glandular or tubular differentiation in the tumor, nuclear pleomorphism, and mitotic rate in assigning the grade of the invasive carcinoma [17].

The molecular classification of breast cancers is as follows: luminal A: the majority are ER-positive, HER2-negative tumors with a low Ki67 index; luminal B: the majority are ER-positive, HER2-positive tumors with a high Ki67 index; HER2-enriched ER-negative tumors with HER2 overexpression; and basal-like tumors with ER and HER2 negativity.

The predominance of invasive carcinoma, no special type among all malignant lesions of the breast, frequent grade II tumors, and immunohistochemical findings observed in this study are comparable with similar studies like Sung Hyun Kim et al. and Suraini Mohamad Saini et al. [18,19]. Claudin-low (CL) tumors are usually ER, PR, and HER2 negative and carry a poor prognosis [1]. PDL-1-positive triple-negative cancers benefit from pembrolizumab as adjuvant chemotherapy [20].

The ER-positive tumors show a good prognosis with hormone therapy (aromatase inhibitors), and the previously prognostically poor HER2-positive tumors are showing a good response to trastuzumab and lapatinib [21]. The International Ki67 in Breast Cancer Working Group (IKWG, 2019) states that the Ki67 index of <5% is categorized as low and >30% is categorized as high. Tumors with a Ki67 index between 5% and 30% must undergo multi-parameter gene expression assays to ascertain their risk. The Ki67 index is particularly useful in ER-positive and HER2-negative tumors to ascertain the need for adjuvant chemotherapy [22]. Patients with a Ki67 index of more than 20% are at high risk for recurrence [20].

The margin status of breast conservative surgery or MRM specimens with at least 1 mm clearance reduced the incidence of local and distant recurrence [23].

BRCA1 and BRCA2 are the most common mutated genes seen in patients with ovarian and breast carcinomas. TP53, PTEN, CDH1, MLH1, MSH2, MSH6, PMS2, and PALB2 are the other mutations in the panel for concurrent ovarian and breast carcinomas [24].

In MRM specimens post-neoadjuvant chemotherapy, the Miller-Payne system for assessing treatment response was applied. The grades include grade 1 (no change or response to chemotherapy), grade 2 (up to 30% loss of tumor cells); grade 3 (between 30% and 90% reduction in tumor cells), grade 4 (marked response with >90% loss of tumor cells), and grade 5 (no malignant cells were identifiable; DCIS may be present). Other criteria, like the residual cancer burden (RCB) index and treatment effects by CAP protocols, are also in use [25].

There were 40 cases of core needle biopsies during the study period with an inconclusive diagnosis. This was due to a sampling error. This points out the need for an ultrasound-guided core needle biopsy and a minimum of six cores to reduce such errors [26]. These cases were not included in the study. BI-RADS 4 and 5 need to be followed up immediately with ultrasound-guided core needle biopsy to rule out or confirm the possibility of malignancy.

A PET-CT scan is useful in identifying nodal involvement and distant metastasis in locally advanced breast cancer. Due to its high cost and low sensitivity to detect small lesions (<1 cm), it is not recommended in T1, 2, or 3 tumors [27].

Limitations

The FNAC diagnosis, core needle biopsy diagnosis, and excision diagnosis are not uniformly available for all the cases in the study, as the diagnostic modality is chosen as per clinical need.

## Conclusions

The sequence of events in the workup of a breast lump, irrespective of age, includes a clinical examination followed by an ultrasonogram or mammogram and a pathological examination (FNAC or core needle biopsy) for a definite diagnosis. Accurately diagnosing breast lesions helps in choosing the right management for the patient and alleviates unnecessary worry for the patient. Imaging modalities (ultrasonogram or mammogram) have good sensitivity and specificity in diagnosing breast lesions and can be reliably used as a preliminary test in breast lump evaluation. In discordant cases, a repeated core needle biopsy or excision has to be recommended, as pathological diagnosis is the cornerstone of effective management. Our institute’s correlation between imaging and pathological diagnosis was comparable with other similar studies.
